# Supplementary material for: Comparative genomics provides new insights into the diversity, physiology, and sexuality of the only industrially exploited tremellomycete: Phaffia rhodozyma
Source: BMC Genomics. 2016 Nov 9;17:901. doi: 10.1186/s12864-016-3244-7 (PMC5103461; doi:10.1186/s12864-016-3244-7)
Supplement: Additional file 6: — List of orphan genes with links to PFAM (related to Additional file 1: Table S1). (ZIP 1428 kb) [file 12864_2016_3244_MOESM6_ESM.zip › PFAM_UNIQUE/G02906_P.html]

#                                                                            --- full sequence --- -------------- this domain -------------   hmm coord   ali coord   env coord
  
# target name        accession   tlen query name           accession   qlen   E-value  score  bias   #  of  c-Evalue  i-Evalue  score  bias  from    to  from    to  from    to  acc description of target
  
#------------------- ---------- ----- -------------------- ---------- ----- --------- ------ ----- --- --- --------- --------- ------ ----- ----- ----- ----- ----- ----- ----- ---- ---------------------
  
G02906\_P             -           1146 DUF1053              PF06327.9    101      0.11   11.6   2.4   1   2    0.0078        47    3.1   0.4    29    65   973  1029   939  1042 0.80 - Domain of Unknown Function (DUF1053)  
G02906\_P             -           1146 DUF1053              PF06327.9    101      0.11   11.6   2.4   2   2   0.00036       2.2    7.4   0.1    16    66  1058  1107  1052  1123 0.82 - Domain of Unknown Function (DUF1053)  
G02906\_P             -           1146 DUF4611              PF15387.1     96      0.79    8.6   6.5   1   2       6.1   1.6e+03   -2.0   0.2    63    79   820   836   800   846 0.63 - Domain of unknown function (DUF4611)  
G02906\_P             -           1146 DUF4611              PF15387.1     96      0.79    8.6   6.5   2   2   0.00071      0.19   10.6   2.1    25    82   960  1020   947  1031 0.70 - Domain of unknown function (DUF4611)  
G02906\_P             -           1146 Nucleoplasmin        PF03066.10   149     0.026   12.7   1.5   1   2       9.4   8.5e+02   -1.9   0.0   121   137   818   834   787   840 0.62 - Nucleoplasmin  
G02906\_P             -           1146 Nucleoplasmin        PF03066.10   149     0.026   12.7   1.5   2   2   0.00067     0.061   11.5   0.6   110   134   996  1020   891  1031 0.70 - Nucleoplasmin  
G02906\_P             -           1146 Ribosomal\_60s        PF00428.14    88       3.1    6.9   7.0   1   1     0.032       7.4    5.7   7.0    59    81   999  1022   983  1028 0.57 - 60s Acidic ribosomal protein
